# 2879. Utilization of vector autoregressive models for multivariable time-series analysis to evaluate the difference between days of therapy and days of antibiotic spectrum coverage in an inpatient antimicrobial stewardship program

**DOI:** 10.1093/ofid/ofad500.156

**Published:** 2023-11-27

**Authors:** Shutaro Murakami, Hitoshi Honda, Manabu Akazawa

**Affiliations:** Tokyo Metropolitan Tama Medical Center, Fuchushi, Tokyo, Japan; Fujita Health University School of Medicine , Toyoake, Aichi, Japan; Meiji Pharmaceutical University, Kiyose-shi, Tokyo, Japan

## Abstract

**Background:**

Days of therapy (DOT), commonly used to estimate antimicrobial consumption, has some limitations. Days of antibiotic spectrum coverage (DASC), a novel metric, overcomes these limitations. This study examined the difference between these two metrics of inpatient intravenous antimicrobial consumption in assessing antimicrobial stewardship efficacy and antimicrobial resistance by using the vector autoregressive (VAR) model with time-series analysis, which has been used in macroeconomics.

**Methods:**

Differences between DOT and DASC were investigated at a tertiary care center in Japan over eight years using VAR models with three variables in the following order: 1) the monthly proportion of prospective audit and feedback (PAF) acceptance as an index of antimicrobial stewardship efficacy; 2) monthly DOT and DASC adjusted by 1,000 days present as indices of antimicrobial consumption; and 3) the monthly incidence of five, drug-resistant organisms as an index of antimicrobial resistance (*Clostridioides difficile* infections (CDI), extended-spectrum β-lactamase (ESBL)-producing Enterobacterales, methicillin-resistant *Staphylococcus aureus* (MRSA), drug-resistant *Pseudomonas aeruginosa,* and *drug-resistant Enterobacterales*).

**Results:**

The Granger-causality test, which evaluates whether incorporating lagged variables can help to predict other variables, found that PAF acceptance contributed to DOT and DASC; these in turn contributed to the incidence of drug-resistant *P. aeruginosa*. Notably, only DASC helped to predict the incidence of drug-resistant Enterobacterales. Another VAR analysis demonstrated that a high proportion of PAF acceptances was accompanied by decreased DASC in a given month while increased DASC was accompanied by an increased incidence of drug-resistant Enterobacterales, unlike with DOT.

**Figure:**
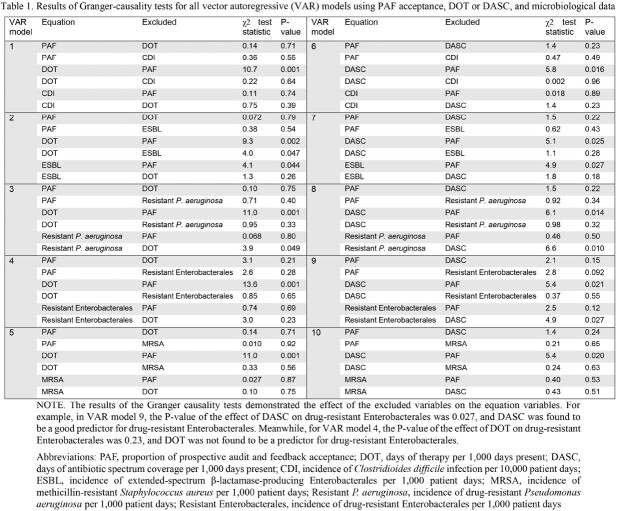

**Conclusion:**

VAR models with variables in the order of PAF acceptance, antimicrobial consumption, and antimicrobial resistance suggested that DASC, including antimicrobial spectrum information, may more accurately reflect the impact of PAF on antimicrobial consumption and be superior to DOT for predicting the incidence of drug-resistant Enterobacterales.

**Disclosures:**

**Hitoshi Honda, MD**, Moderna: Honoraria **Manabu Akazawa, MPH, PhD**, Astellas Pharma: Advisor/Consultant|GSK: Advisor/Consultant|Jansen: Advisor/Consultant|Shionogi: Advisor/Consultant|Takeda: Advisor/Consultant

